# Association of hypoxia-inducible factor-1α (HIF1α) 1790G/A gene polymorphism with renal cell carcinoma and prostate cancer susceptibility: a meta-analysis

**DOI:** 10.1186/s12881-019-0874-z

**Published:** 2019-08-16

**Authors:** Hong-Yan Li, Tianbiao Zhou, Wenshan Lin, Shujun Lin, Hongzhen Zhong

**Affiliations:** 1 Department of Nephrology, Huadu District People’s Hospital of Guangzhou, Southern Medical University, Guangzhou, 510800 China; 2 Department of Nephrology, the Second Affiliated Hospital of Shantou University Medical College, 515041, No 69 Dongxia Road, Shantou, China

**Keywords:** Renal cell carcinoma, Prostate cancer, Hypoxia-inducible factor-1α (HIF1α), 1790G/A gene polymorphism, Meta-analysis

## Abstract

**Background:**

This meta-analysis was performed to evaluate the relationship between hypoxia-inducible factor-1α (HIF1α) 1790G/A gene polymorphism and the susceptibility to renal cell carcinoma (RCC) and prostate cancer (PCa).

**Methods:**

Association investigations were identified and included from the Embase, Cochrane Library and PubMed databases on March 1, 2018, and eligible investigations were analyzed by meta-analysis. Odds ratios (OR) were used to express the dichotomous data, and the 95% confidence intervals (CI) were also calculated.

**Results:**

In this meta-analysis, we found that the AA genotype of HIF1α 1790G/A was positively associated with the risk of RCC in overall populations, Caucasians, but not for Asians. G allele and GG genotype were not associated with the susceptibility of RCC in overall populations, Caucasians, and Asians. The G allele was negatively associated with PCa susceptibility in overall populations, Asians, but not for Caucasians. GG genotype was negatively associated with PCa susceptibility in Asians, but not for overall populations and Caucasians. HIF1α 1790G/A AA genotype was not associated with PCa susceptibility in overall populations of Caucasians or Asians.

**Conclusion:**

AA genotype of HIF1α 1790G/A was positively associated with RCC risk in overall populations and Caucasians. Furthermore, the G allele was negatively associated with prostate cancer susceptibility in overall populations, Asians, and GG genotype was negatively associated with PCa susceptibility in Asians.

## Background

Renal cell carcinoma (RCC) is one of the most commonly occurring types of tumors in the urogenital system and accounts for ~ 85% of all kidney tumors [1–4]. RCC is not sensitive to conventional chemotherapy and radiotherapy, and its prognosis remains poor [1]. Prostate cancer (PCa) is a complex disease, and is the fifth leading cause of cancer death in men worldwide [5]. The screening projection for PCa is still unclear and recent large clinical trials have failed to exert notable reduction in the prostate-specific mortality and the all-cause mortality [6]. The current evidence shows that RCC and PCa are Von Hippel-Lindau tumor suppressor (VHL)-related cancers [7–10]. VHL protein is an E3 ubiquitin ligase that targets hypoxia inducible factor 1α (HIF1α) to the proteasome for degradation [11]. The current literature indicates that genetic factors are significant contributors to cancers risk [12–15].

Hypoxia inducible factor 1α (HIF1α) is the central regulator of the cellular response to low oxygen, and the activity of HIF1α is down-regulated in various human pathologies [16, 17]. During hypoxia, reduced oxygen availability can inhibit these HIF hydroxylase enzymes, and lead to HIF1α protein accumulation, which may translocate to the cell nucleus, bind to the HIF1β, and induce the transcription of some HIF target genes [18]. HIF1α regulates tumor cell proliferation, invasion, migration, and resistance to radiotherapy [16, 19]. Consequently, given the importance of HIF signaling in disease, there is considerable interest in developing strategies to modulate HIF1α activity and to induce down-stream signaling events. HIF1α 1790G/A (rs11549467) gene polymorphism is one of the most important gene polymorphism for certain cancers, such as PCa, and RCC. However, the available evidence is inadequate due to inconsistencies between studies and an overall lack of data. This meta-analysis was performed to investigate whether the HIF1α 1790G/A (rs11549467) gene polymorphism is associated with RCC, PCa susceptibility.

## Methods

### Search strategy

Retrieval of the relevant published reports were conducted in the electronic databases of Embase, Cochrane Library and PubMed on March 1, 2018, and eligible original articles were recruited into this meta-analysis. The key phrases for retrieval consisted of (“hypoxia-inducible factor OR hypoxia-inducible factor-1α” OR “HIF1α”) and (“renal cell carcinoma” OR “renal carcinoma” OR “renal cancer” OR “RCC” OR “prostatic carcinoma” OR “prostatic cancer” OR “prostate cancer” OR “prostate carcinoma”).

### Inclusion and exclusion criteria

**Inclusion criteria:** (1) an endpoint of RCC, PCa; (2) two comparison groups (case vs. control); (3) the presence of detailed data for the genotype distribution.

**Exclusion criteria:** (1) case reports, review articles and editorials; (2) preliminary results not on HIF1α 1790G/A gene polymorphism or RCC, PCa susceptibility; (3) investigations of the role HIF1α gene expression in disease.

### Data extraction and synthesis

The following information from each eligible study was independently extracted by two investigators: first author’s surname, year of publication and the number of cases and controls for HIF1α 1790G/A genotypes. Frequencies of genotypes for HIF1α 1790G/A were calculated for each case group and control group, from the corresponding genotype distributions. The results were compared, and disagreement was resolved by discussion. Consistency of data extracted by the 2 researchers was evaluated and disagreements were resolved by discussion.

### Statistical analysis

All statistical analyses were performed using Cochrane Review Manager Version 5 (Cochrane Library, UK). The pooled statistic was determined using the fixed effects model (Mantel-Haenszel method), and a random effects model (DerSimonian-Laird method) was conducted when the *P-*value from the heterogeneity test was less than 0.1. Odds ratios (OR) were used to express the dichotomous data, and 95% confidence intervals (CI) were also calculated. A *P* < 0.05 was required for the pooled OR to be statistically significant. *I*^*2*^ was used to assess the heterogeneity among the included studies.

## Results

### Study characteristics for association of the HIF1α 1790G/A gene polymorphism with RCC susceptibility

Four studies [20–23] were recruited into our investigation of the relationship between the HIF1α 1790G/A gene polymorphism and RCC susceptibility (Table 1). Data of interest was extracted by the first author’s surname, year of publication and the number of cases and controls for the HIF1α 1790G/A genotype (Table 1). The 4 included investigations contained 1139 case series and 1364 controls.

**Table 1:** Characteristics of studies evaluating the effects of hypoxia-inducible factor-1α (HIF1α) 1790G/A gene polymorphism on renal cancer and prostate cancer risk

Cancer Types	Author, Year	Country	Ethnicity	Case			Control		
				–	+	Total	–	+	Total
Renal cancer	Clifford 2001	UK	Caucasian	18	27	45	31	17	48
	Ollerenshaw 2004	UK	Caucasian	89	84	173	117	94	211
	Morris 2009	Poland	Caucasian	63	63	126	255	250	505
	Qin 2012	China	Asian	50	50	100	108	92	200
Prostate cancer	Chau 2005	USA	Mix	424	487	911	555	677	1232
	Orr-Urtreger 2007	Israel	Caucasian	51	47	98	167	157	324
	Li 2007	USA	Mix	303	321	624	433	454	887
	Li 2012	China	Asian	46	30	76	86	96	182

### Study characteristics for association of the HIF1α 1790G/A gene polymorphism with PCa susceptibility

Four studies [24–27] were recruited into our meta-analysis to explore the relationship between the HIF1α 1790G/A gene polymorphism and PCa risk (Table 1). Those four investigations contained 2124 case series and 2476 controls.

### Association of the HIF1α 1790G/A gene polymorphism with RCC susceptibility

**Fig. 1:**
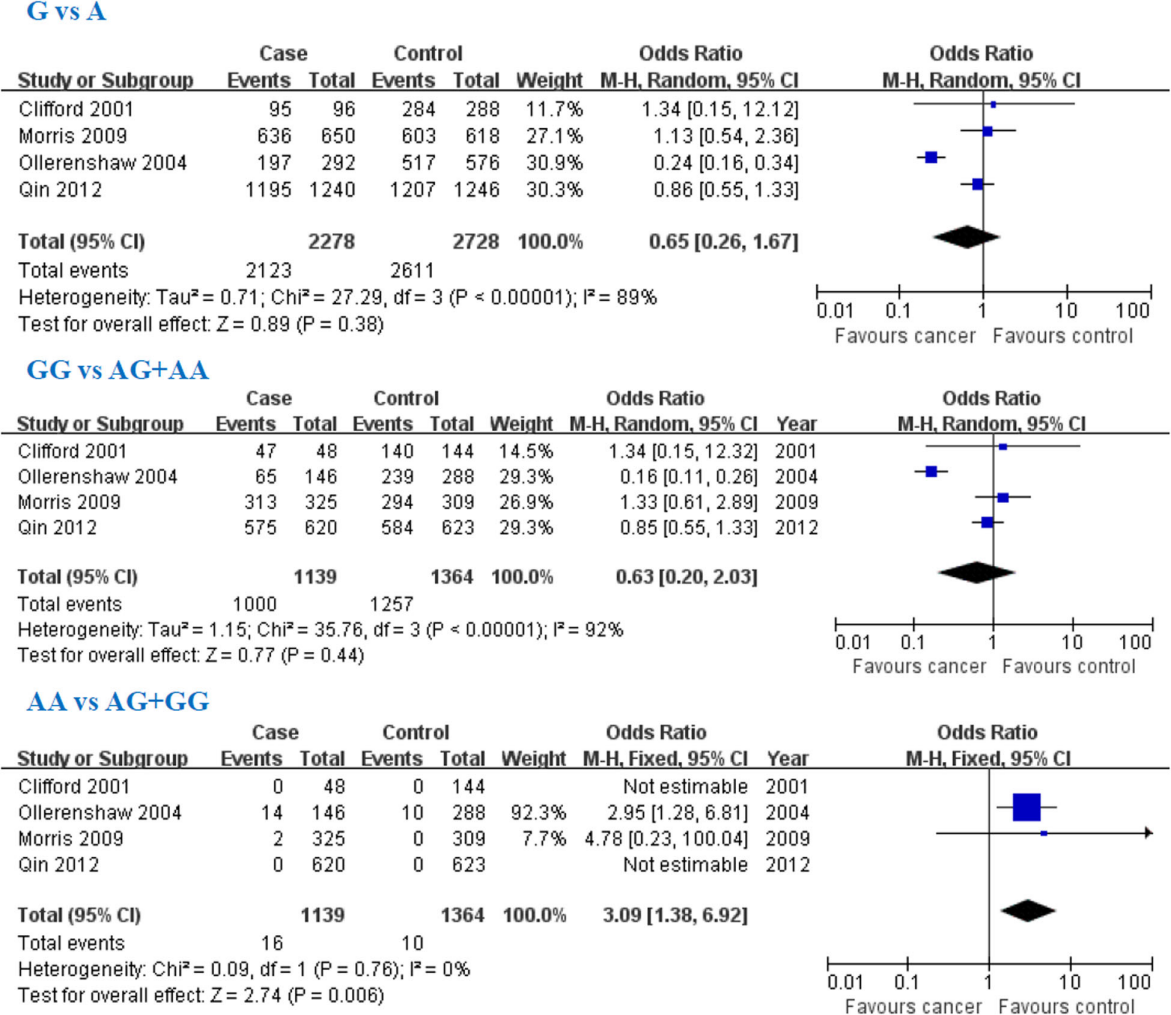
Association between hypoxia-inducible factor-1α (HIF1α) 1790G/A gene polymorphism and renal cancer susceptibility in overall populations

In this meta-analysis, we found that the AA genotype of HIF1α 1790G/A was positively associated with RCC risk in overall populations (OR = 3.09, 95% CI: 1.38–6.92, *P* = 0.006; Fig. 1 and Table 2) and Caucasians, but not for Asians. G allele and GG genotype were not associated with RCC risk in overall populations (G: OR = 0.65, 95% CI: 0.26–1.67, *P* = 0.38; GG: OR = 0.63, 95% CI: 0.20–2.03, *P* = 0.44; Fig. 1 and Table 2), Caucasians, or Asians.

**Table 2:** Meta-analysis of the association of hypoxia-inducible factor-1α (HIF1α) 1790G/A gene polymorphism with renal cancer and prostate cancer

Cancer Type	Group and subgroups	Studies Number	Q test *P*-value	Model selected	OR (95%CI)	P
Renal cancer						
G vs A	Overall	4	<0.00001	Random	0.65 (0.26,1.67)	0.38
	Caucasian	3	0.0004	Random	0.61 (0.16,2.30)	0.46
	Asian	1	–	Fixed	0.86 (0.55,1.33)	0.49
AA vs AG + GG	Overall	4	0.76	Fixed	3.09 (1.38,6.92)	0.006
	Caucasian	3	0.76	Fixed	3.09 (1.38,6.92)	0.006
	Asian	1	–	Fixed	–	–
GG vs AG + AA	Overall	4	<0.00001	Random	0.63 (0.20,2.03)	0.44
	Caucasian	3	<0.00001	Random	0.59 (0.11,3.31)	0.55
	Asian	1	–	Fixed	0.85 (0.55,1.33)	0.48
Prostate cancer						
G vs A	Overall	4	0.49	Fixed	0.68 (0.47,0.99)	0.04
	Caucasian	1	–	Fixed	0.67 (0.09,4.74)	0.68
	Asian	1	–	Fixed	0.58 (0.36,0.91)	0.02
AA vs AG + GG	Overall	4	–	Fixed	3.25 (0.13,79.90)	0.47
	Caucasian	1	–	Fixed	–	–
	Asian	1	–	Fixed	3.25 (0.13,79.90)	0.47
GG vs AG + AA	Overall	4	0.50	Fixed	0.69 (0.47,1.00)	0.05
	Caucasian	1	–	Fixed	0.66 (0.09,4.76)	0.68
	Asian	1	–	Fixed	0.58 (0.36,0.92)	0.02

### Association of the HIF1α 1790G/A gene polymorphism with PCa susceptibility

**Fig. 2:**
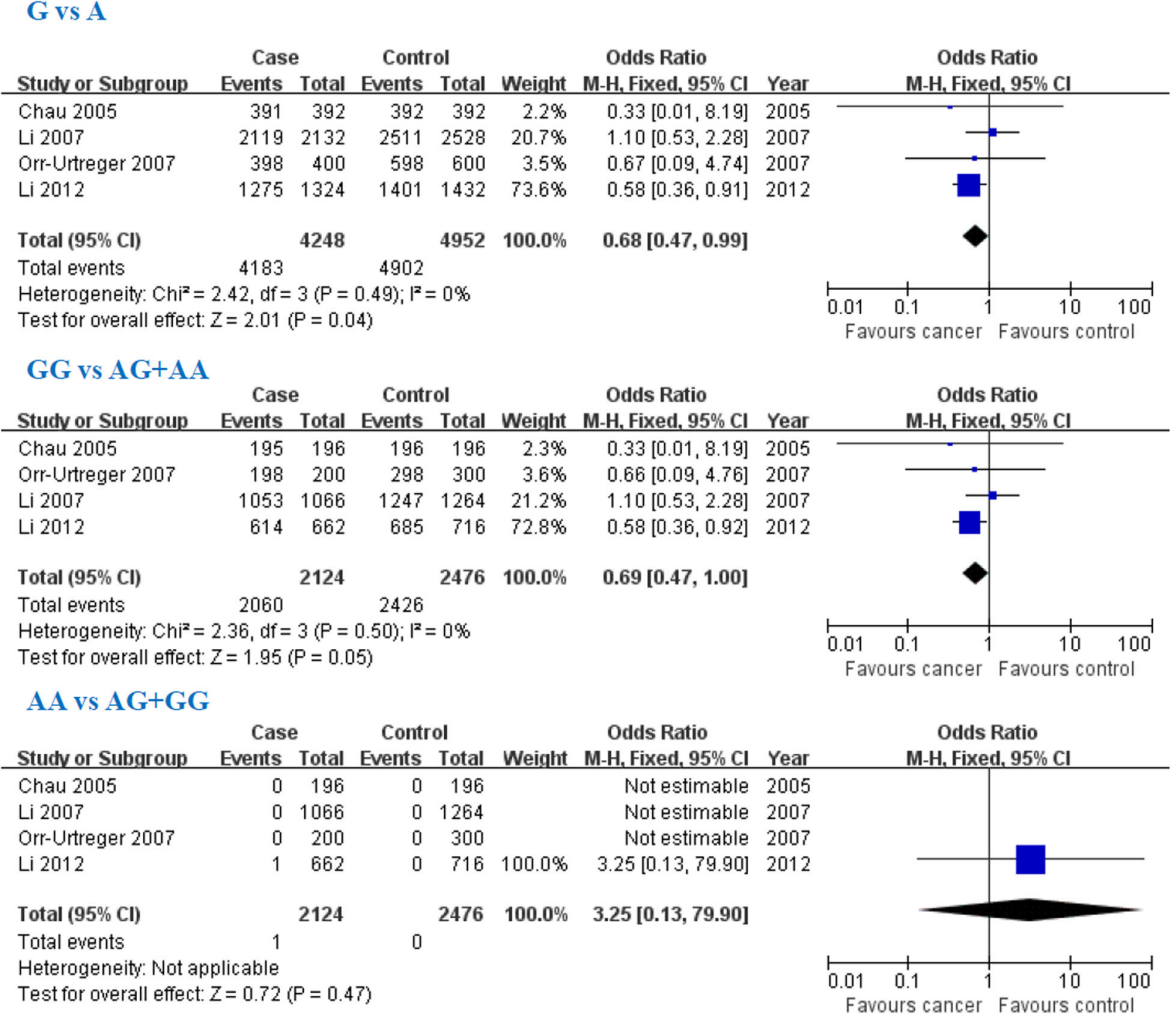
Association between hypoxia-inducible factor-1α (HIF1α) 1790G/A gene polymorphism and prostate cancer susceptibility in overall populations

The G allele was negatively associated with PCa susceptibility in overall populations and Asians, but not for Caucasians (Overall populations: G: OR = 0.68, 95% CI: 0.47–0.99, *P* = 0.04; Fig. 2 and Table 2). GG genotype was negatively associated with PCa susceptibility in Asians, but not for overall populations or Caucasians (Overall populations: G: OR = 0.69, 95% CI: 0.47–1.00, *P* = 0.05; Fig. 2 and Table 2). However, HIF1α 1790G/A AA genotype was not associated with PCa susceptibility in overall populations of Caucasians or Asians (Overall populations: OR = 3.25, 95% CI: 0.13–79.90, *P* = 0.47; Fig. 2 and Table 2).

## Discussion

In this in-depth meta-analysis, the results indicate that the AA genotype of HIF1α 1790G/A is positively associated with the risk of RCC in overall populations and Caucasians, but not for Asians. The G allele and GG genotype are not associated with the susceptibility of RCC in overall populations, Caucasians, and Asians. The G allele is negatively associated with PCa susceptibility in overall populations and Asians, but not for Caucasians. The GG genotype is negatively associated with PCa susceptibility in Asians, but not for overall populations or Caucasians. However, the HIF1α 1790G/A AA genotype is not associated with PCa susceptibility in overall populations, Caucasians or Asians.

Previous, related meta-analyses have been conducted. Li et al. [28] reported that for the 1790G/A polymorphism, the G allele was significantly associated with a higher risk of urinary cancers in Asians. Anam et al. [29] conducted a meta-analysis using the genome wide association method including 19 case-control studies with a total sample size 10,654, and reported that the HIF1α 1790 G/A gene polymorphism significantly increased the risk of cancer in both Asian and Caucasian populations. Zhou et al. [30] performed a meta-analysis of 28 case-control studies of the relationship between the HIF1α G1790A gene polymorphism and the risk of cancer, and reported that the G with A of HIF-1α G1790A gene polymorphism is a notable risk factor of cancer, especially for RCC, lung cancer, pancreatic cancer, and head and neck cancer. These meta-analyses did not assess the relationship between the HIF1α 1790G/A gene polymorphism and RCC and PCa susceptibility by races. Our results indicate that AA genotype of HIF1α 1790G/A was positively associated with RCC risk in overall populations and Caucasians. Furthermore, the G allele was negatively associated with PCa susceptibility in overall populations and Asians, and the GG genotype was negatively associated with PCa susceptibility in Asians. However, the sample sizes were small and these results need to be treated with caution.

Considering our results and the available literature, we suspected that the G allele and GG genotype were negatively associated with PCa susceptibility, and that the AA genotype was a risk factor to induce the onset of RCC. The hypothesis was as follows: Under both normoxic conditions and hypoxia, the HIF-1α G1790A gene polymorphism would be associated with increased transcription activities and enhanced angiogenesis compared to the wild type [31, 32]. The cause of the enhance transactivation capacity could be the alteration of stability of variant proteins or the enhanced recruitment of transcriptional co-factors such as SRC-1 and CBP/p300 that interact with HIF-1α [33]. HIF-1α G1790A gene polymorphism has been detected within the oxygen-dependent degradation/pVHL binding domain in exon 12 of the HIF-1α gene, which induces increased transcription activity compared to wild type [31]. In addition, regulatory region mutations may interfere with different post-translational modifications of HIF-1α and result in enhanced protein stability [34, 35]. HIF-1α G1790A has been associated with enhanced tumor-produced HIF-1α and cancer progression [36]. BHLHE41 is a specific transcriptional target of HIF-1α, and the HIF-1α G1790A polymorphism creates a HIF-binding site to mediate the upregulation of BHLHE41 [37]. However, there was rare study to detect the functional roles of the G, GG, and AA genotypes in the transcription and other related activities of HIF-1α. In this study, we found that the negative association of G allele with susceptibility of prostate cancer in Asians. We suspected that G allele of HIF-1α G1790A might be associated with low levels of HIF-1α and might be negative association of G allele with prostate cancer risk. However, more studies should be performed to confirm it.

There were some limitations in our meta-analysis, as the study sample sizes were low, and we did not explore sources of variability between studies such as adjusting variables, age distribution, and sources of controls. These results should be treated with caution.

## Conclusion

The present results support the hypothesis that the AA genotype of HIF1α 1790G/A is positively associated with RCC risk in overall populations and Caucasians. Furthermore, the G allele is negatively associated with PCa susceptibility in overall populations and Asians, and the GG genotype is negatively associated with PCa susceptibility in Asians. However, additional investigations are required to confirm these relationships.

## Data Availability

The datasets used and/or analyzed during the current study are available from the corresponding author on reasonable request.

## Abbreviations

CI: confidence intervals
HIF1α: hypoxia-inducible factor-1α
OR: Odds ratios
PCa: prostate cancer
RCC: renal cell carcinoma
